# Effects of Fourteen-Day Bed Rest on Trunk Stabilizing Functions in Aging Adults

**DOI:** 10.1155/2015/309386

**Published:** 2015-10-25

**Authors:** Nejc Sarabon, Jernej Rosker

**Affiliations:** ^1^Department for Health Study, Andrej Marušič Institute, University of Primorska, Muzejski Trg 2, SI-6000 Koper, Slovenia; ^2^Faculty of Mathematics, Natural Sciences and Information Technologies, University of Primorska, Glagoljaska 8, SI-6000 Koper, Slovenia; ^3^S2P Ltd., Laboratory for Motor Control and Motor Behaviour, Tehnoloski Park 19, SI-1000 Ljubljana, Slovenia

## Abstract

Bed rest has been shown to have detrimental effects on structural and functional characteristics of the trunk muscles, possibly affecting trunk and spinal stability. This is especially important in populations such as aging adults with often altered trunk stabilizing functions. This study examined the effects of a fourteen-day bed rest on anticipatory postural adjustments and postural reflex responses of the abdominal wall and back muscles in sixteen adult men. Postural activation of trunk muscles was measured using voluntary quick arm movement and sudden arm loading paradigm. Measurements were conducted prior to the bed rest, immediately after, and fourteen days after the bed rest. Immediately after the bed rest, latencies of anticipatory postural adjustments showed significant shortening, especially for the obliquus internus and externus muscles. After a fourteen-day recuperation period, anticipatory postural adjustments reached a near to complete recovery. On the contrary, reactive response latencies increased from pre-bed-rest to both post-bed-rest measurement sessions. Results indicate an important effect of bed rest on stabilizing functions of the trunk muscles in elderly adults. Moreover, there proved to be a significant deterioration of postural reactive responses that outlasted the 14-day post-bed-rest rehabilitation.

## 1. Introduction

Trunk and spinal stability have been proposed as an important factor for preventing spinal injuries and disease [1, 2]. Passive (i.e., skeletal and connective tissue system), active (i.e., muscular system), and neural (i.e., central and peripheral nervous system) systems have been proposed to be fundamental for preserving posture and stabilizing the spine [3]. Hoffman and Gabel [2] extended this model arguing that a stable spine is important for effective and injury-free movements of the limbs. For more than twenty years different training intervention strategies have emerged, focusing on improving spinal and trunk stability [4, 5]. On the other hand, new insights into changes in spinal and trunk stability following injury or disease are available. However, much less is known about the alterations in spinal and trunk stability following long term inactivity (i.e., bed ridden medical intervention) [6]. This is especially important for more vulnerable populations such as aging adults who are more prone to medical issues demanding hospitalisation [6, 7].

For studying the ability of the trunk to maintain stability during anticipated perturbations, a quick arm movement paradigm has been used. In this type of movement the neural controlling centres anticipate the extent to which posture will be perturbed and initiate compensatory actions prior to the perturbation onset (i.e., anticipatory postural adjustments or APAs). As in conditions of unanticipated perturbations, the sudden arm loading paradigm has been used. These responses to perturbation are primarily reflexive/reactive in their nature (i.e., reflex responses or RRs). The magnitude of stability loss has been quantified using biomechanical parameters (i.e., centre of body mass (CoM) and centre of pressure (CoP)) [8] and electromyographic parameters (i.e., latency and magnitude of the EMG) [9, 10]. The electromyographic responses have been shown to be correlated with the CoP amplitude as muscle activation tunes the body and the trunk stiffness consequently affecting the body sway measured via CoP and CoM [8, 10].

Changes in postural and trunk-spinal stability have been well documented in aging adults [10–12]. Their reactions to postural perturbations change from distal to proximal, suggesting an increased dependence on the hip strategy [13]. Moreover, the RRs of the lower limb and trunk muscles to unanticipated perturbations are delayed when compared to their younger peers [14]. The APAs have been shown to be altered but generally preserved, suggesting less robust stabilization strategies [12]. As suggested by the literature more profound changes have been observed for RRs than for APAs [14].

In addition to the functional changes, aging adults often suffer from medical issues demanding prolonged bed rest (BR). To our knowledge, no studies have been done that have addressed the effects of prolonged BR on the neuromuscular aspects of spinal (i.e., trunk) stability. The majority of the BR studies have been completed on young healthy adults indicating significant anatomical changes (i.e., decrease in trunk muscle cross-sectional area [15–17] and changes in spinal morphology [18]) and functional changes (shift from tonic to phasic activity of the lower back muscles [19, 20] and changes in body sway [21]). These changes have been shown to persist for at least 56 days after a prolonged BR [16, 17]. These findings suggest that the active and neural subsystems for spine stabilization could be affected by prolonged BR and cause changes in APAs and RRs.

The purpose of this study was to assess changes in APAs and RRs latencies of the trunk stabilizing muscles following a fourteen-day BR in aging adults. In addition we wanted to assess the recuperation of APAs and RRs after a fourteen-day-long post-BR phase (including a training intervention). Our first hypothesis states that the onset of APAs will be closer to the activation of the prime movement muscle and RRs would be significantly delayed following the fourteen days of BR. Our second hypothesis states that in the post-BR recuperation phase these changes would reverse to pre-bed-rest levels for both APAs and RRs.

## 2. Materials and Methods

### 2.1. Participants

Sixteen healthy men ([mean (standard error)] of 59.6 (3.4) years and with body height of 173.3 (4.9) cm, body mass 77.3 (11.8) kg, and BMI 25.8 (3.7) kg/m^2^) that were recruited from the local community volunteered in the study. Prior to the enrolment, each participant underwent a medical evaluation. The following were used as exclusion criteria: diabetes, active malignancy, uncontrolled hypertension, history of cardiovascular disease, history of deep vein thrombosis/pulmonary embolism, significant hepatic or renal disease, chronic inflammatory disease, any significant impairment of the locomotor system, and vestibular or uncorrected visual disturbance. Prior to the enrolment each participant was informed of the protocol and potential risks of the study and was required to sign a written informed consent, confirmed by the Slovenian National Committee for Medical Ethics. All procedures were in accordance with the Declaration of Helsinki and Oviedo Convention.

### 2.2. Study Protocol

During the 14 days of horizontal BR without physical countermeasures, participants were required to restrict physical activity and reduce deviations from the horizontal lying position to a minimum, also during showering and toileting. Following BR, participants completed 2 weeks of a rehabilitation protocol (three times per week). A typical session consisted of a warm-up (6 min of low intensity Nordic walking and 6 min of active stretching), main part (4 balance exercises, 6 strength exercises, and Nordic walking endurance protocol), and cooldown (simple breathing exercises). For more information on the Valdoltra BR study and rehabilitation protocol the reader is directed to the work by Goswami and colleagues [22]. Follow-up measurements were performed 14 days after the end of the BR period. Participants were evaluated (i) before the BR (PRE), (ii) immediately after the BR (POST0), and, finally, (iii) 14 days after the BR (POST14). During the assessment protocol, participants were evaluated with the measurement protocol for the evaluation of trunk postural (pre/re)actions as described below.

### 2.3. Measurement Tasks and Procedures

Each of the evaluations consisted of two tests: measurements of APAs and measurements of RRs to sudden loading. At the beginning, a 5-minute standardized warm-up was performed (spot running with high knees 2.5 min, 10 squats, and 10 push-ups with hands supported on the wall). Before undertaking each test, participants performed five introductory trials. Participants were barefoot and asked to place their feet at the hip-width during all of the measurements. Participants were constantly reminded to maintain their normal posture and to stand relaxed. All trials were triggered in a random manner every 5 to 12 s with 24 repetitions in total for each test (3 sets × 8 repetitions, 1 min breaks).

**Figure 1:**
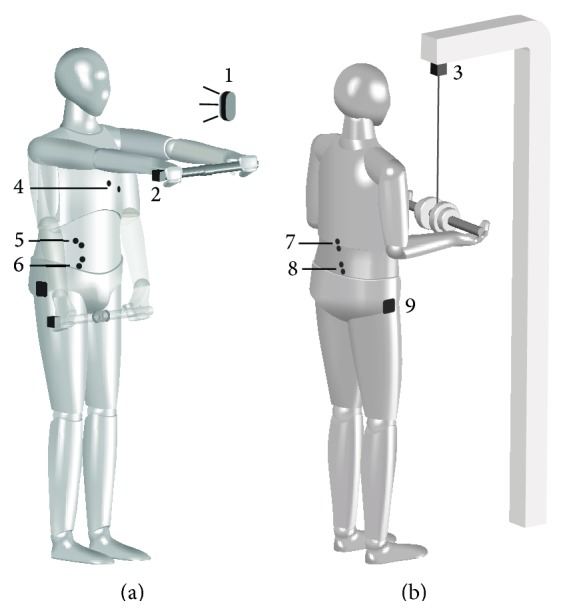
Presentation of fast bilateral shoulder flexion (a) and sudden arm loading task (b). A detailed positioning of the visual cue (1), accelerometer (2), load release system (3), electrocardiogram electrodes (4) and EMG electrodes for obliquus externus (5), muscle obliquus internus (6), muscle erector spine (7), muscle multifidus (8), and reference electrode (9) is presented.

*Measurements of APAs* were performed on a random visual cue (LED light, eyes high, and 1.5 m distance). The participant stood with his/her arms extended down by their sides and upon the visual signal the task was to raise a 1.2 kg bar as fast as possible with straight arms up to shoulder height, hold the position for ~1 s, and return the bar back down to the starting position slowly (Figure 1(a)) [23, 24]. In* measurements of RRs to sudden loading* participants stood relaxed, with their elbows flexed to 90° and palms slightly touching the weight handle (8% of the individual's body mass, for more detail see Figure 1(b)) [25]. A sudden release of the load was achieved by a custom built electromagnetic mechanism. After load release, the participants' task was to return to and settle at the initial position, as quickly as possible.

The setup was controlled by bespoke software (Labview 2012, National Instruments, Texas, USA), which triggered the visual clues and the quick release mechanism. Triggering was synchronized with an electrocardiogram (QRS-wave + 200 ms) so that the postural activation of the trunk muscles appeared during two consecutive QRS-peaks [26]. This prevented ECG interference [27], which could hamper the analysis of the electromyographic (EMG) signals.

### 2.4. Electromyography

The activity of five trunk muscles was acquired with surface EMG. Signals were 3,000x amplified (Biovision, Wehrheim, Germany), A/D converted, and sampled at 10,000 Hz (USB-6343, National Instruments, Texas, USA). Self-adhesive pairs of electrodes (Blue Sensor N, Ambu A/S, Ballerup, Denmark) were used, placed with 2 cm center-to-center distance. EMG of obliquus externus (EO), multifidus at level L5 (MU), erector spine at level L1 (LE), obliquus internus (OI), and deltoideus anterior (DE) were recorded on the right side of the body. Skin preparation and electrode placement were done according to SENIAM recommendations [28]. As this standard does not include recommendations for abdominal muscles, we followed the electrode placement from previous studies with regard to the external and internal oblique muscles [29–32]. An additional pair of electrodes was placed for ECG detection, with one electrode in the region of xiphoid process of sternum and second on the 1/3 of the left ribcage arc. A reference electrode was positioned on the area of the right greater trochanter.

### 2.5. Signal Processing

**Figure 2:**
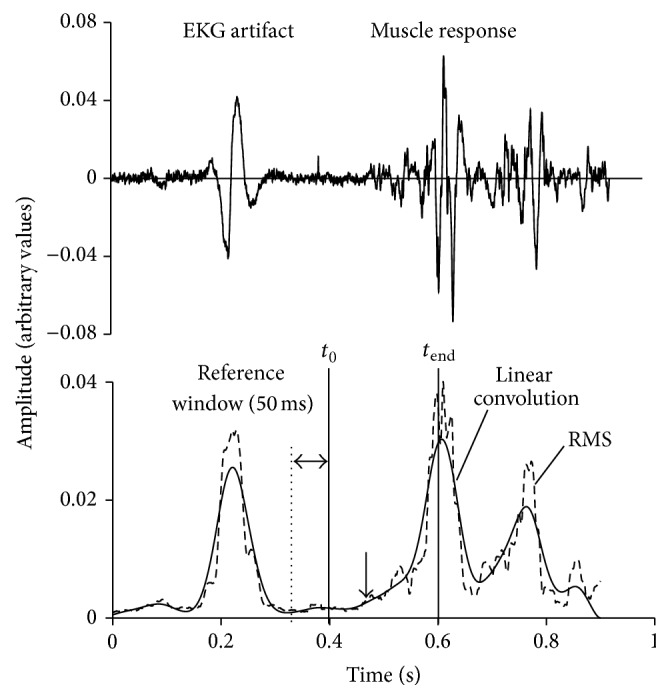
An example of a postural reflex response to sudden loading (muscle obliquus abdominis externus). Instance of load drop (*t*_0_): 200 ms window for analysis (*t*_end_) and 50 ms reference window for calculating baseline EMG activation are shown. The arrow depicts the muscle activation onset, solid line depicts the linear convolution, and the dotted line depicts the root mean square (RMS).

Signals were band-pass filtered (zero lag Butterworth filter 10 Hz/1 kHz, order 2), rectified using a root mean square smoothing filter (window of 20 ms), and low-pass filtered (zero lag Butterworth filter 10 Hz, order 2) to get a linear envelope. Approximated generalized likelihood-ratio step algorithm (AGLRstep) [33] for automatic detection was used to determine the beginning of muscle activity. In APA measurements, activation onset detection was limited with a time window from 200 ms before to 50 ms after the activation of the prime mover muscle (being the DE) [34, 35]. APAs onset times were calculated as the difference between the onset of trunk muscle activation and the activation of the DE muscle. When the activation of the postural muscle preceded the hand movement initiation, the value was negative. In the RRs measurements the activation onset detection was limited to the time window from the moment of the mechanism release (*t*_0_) to 200 ms after (*t*_end_) (Figure 2) [36]. RRs onset times were calculated as a delay from the mechanism release to the trunk muscle activation.

Each muscle was considered active when the processed EMG signal exceeded the average plus 2 standard deviations of the baseline EMG signal [37]. Baseline EMG signal was calculated from the 50 ms reference window directly before the activation onset detection limits, where there was no task related activation or ECG artefacts [37]. All signals were later manually inspected and corrected when activation was incorrectly detected. When there was no activation of the investigated muscle, or activation was not within the detection limits, the trial was not used in further analysis.

### 2.6. Statistical Analysis

Statistical analyses (SPSS 18.0 software, SPSS Inc., Chicago, USA) were performed as follows. Descriptive statistics were calculated for all variables and reported as* mean (standard errors)*. One-way repeated-measures ANOVA were run to test the differences between the testing sessions (PRE, POST0, and POST14). Two-tailed pairwise *t*-tests with Bonferroni corrections were used for pairwise comparisons. The level of statistical significance (*p*) was set at 0.05 and effect size (ES) values were calculated.

## 3. Results

From 24 trials in each task, the following number of trials were removed due to an unrecognizable response (responses not exceeding 2 standard deviations of the baseline): (1) for anticipation task EO: [mean (standard error)] 3.8 (2.4), IO: 3.7 (2.3), LE: 3.6 (2.5), and MU: 3.3 (2.7) and (2) for reaction task EO: 2.1 (2.0), IO: 2.6 (2.6), LE: 2.1 (1.8), and MU: 2.0 (2.0). Latencies in the anticipation task ranged from −22 to −5 ms for the back muscles (LE and MU) and from −3 to 22 ms for the abdominal wall muscles (EO and IO). Similar behaviour was observed for the reaction task where the back muscle latencies ranged between 98 and 108 ms and the abdominal wall muscle latencies ranged between 109 and 122 ms.

**Figure 3:**
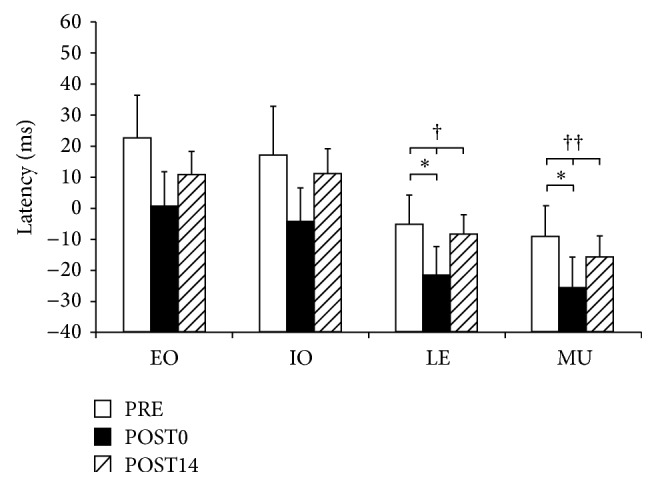
Results for APAs of individual muscles at all three sessions. The statistical significant change assessed by one-way repeated-measures ANOVA is presented by † (*p* < 0.05) and †† (*p* < 0.01). The results of *t*-test are represented by *∗* (*p* < 0.05). Muscles presented are obliquus externus (EO), obliquus internus (OI), erector spinae at level L1 (LE), and multifidus (MU).

APA latencies for all three sessions are presented in Figure 3 and additional details of statistical analysis are presented in Table 1. There were statistically significant differences among all sessions for LE and MU muscle but not for the EO and IO. The decrease in the latency from PRE to POST0 was observed for all muscles. However, only changes in LE muscle and MU muscle were statistically significant. There was a clear increase in the latency from POST0 to POST14 for all muscles, but these changes were not statistically significant (*p* > 0.05). None of the muscles' latencies at POST14 returned completely to the level of PRE. However, differences between these two sessions were not statistically significant (*p* > 0.05) for any of the muscles.

**Figure 4:**
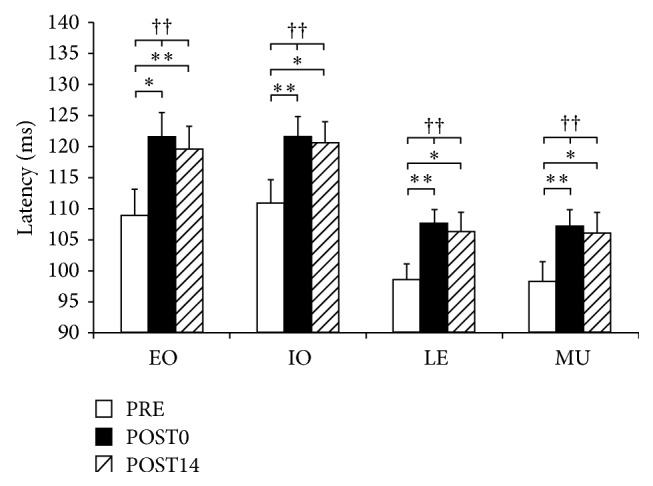
Results for RRs of individual muscles at all three sessions. The statistical significant change assessed by one-way repeated-measures ANOVA is presented by † (*p* < 0.05) and †† (*p* < 0.01). The results of *t*-test are represented by *∗* (*p* < 0.05) and *∗∗* (*p* < 0.01). Muscles presented are obliquus externus (EO), obliquus internus (OI), erector spinae at level L1 (LE), and multifidus (MU).

All muscles showed statistically significant differences (*p* < 0.01) in the reaction latencies among all sessions (Figure 4 and Table 1). An evident increase in the latency from PRE to POST0 was observed for all muscles (*p* < 0.05). A slight decrease in the latency from POST0 to POST14 was observed for all muscles, but these changes were not statistically significant. Consequently, the latency for any muscle at POST14 did not return to the level of PRE. Moreover, statistically significant differences in latency were observed for all muscles (*p* < 0.05).

## 4. Discussion

This study was the first to analyse the effects of a short term BR on the EMG aspects of the APAs and RRs of the trunk muscles in aging adults. As predicted in our first hypothesis, APAs and RRs EMG latencies were changed following fourteen days of inactivity. Interestingly, the trend of the adaptations of the APAs and RRs EMG latencies to BR was not uniform regarding to the different muscle groups. The second hypothesis can only be partially confirmed. The recovery to pre-BR levels was observed only for APAs but not for RRs regardless of the muscle group observed.

The changes in APAs and RRs EMG latencies following BR were expected, as deconditioning of the passive, active, and neural mechanisms was shown to take place during BR. The passive system, especially the ligamentous system, could have probably been affected by changes in spinal curvature (posterior lumbar ligaments are put under additional tension) and changes in the intervertebral discs height and volume as shown in previous studies [18]. These structures are rich in proprioceptors and are possibly responsible for the changes in afferent sensory input to the nervous system [38]. Previous BR studies have also shown significant effect of BR on the active (i.e., muscular) system due to muscle wasting [16, 17]. And finally, changes in muscle activation properties dependent on the activity of the nervous system have also been proposed as indicators of central changes following BR [19, 20].

A trend of earlier muscle EMG onset during APAs was present for abdominal wall and back muscles by observing the group averages. However, only changes in EMG onset for the back muscles were statistically significant. These observations could be partially explained by the results of previous studies, reporting selective changes in the muscle cross-sectional area. Erector spinae, quadratus lumborum, and especially the multifidus muscles have been shown to suffer from the most prominent decreases in the cross-sectional area [15–17], but much less or no atrophy was observed in the abdominal muscles (OE, IO, and rectus abdominis) [16, 17]. Central inhibition usually accompanies muscle inactivity-immobilization [39] possibly explaining the central mechanisms of delayed EMG onset during APAs in more affected muscle groups. An additional cause could be the specific adaptation of the intramuscular coordination during APAs. In the elderly these responses have been shown to be less uniform and include more pronounced changes in proximal stabilization strategies [10, 12]. However further research is needed to assess these possible changes in aging adults following longer periods of inactivity. Based on the reports, showing APA sensitivity to aging and BR to a lesser extent than RRs [14], APAs can be considered as a robust trunk and spine stabilizing strategy. However, future research should include observations of EMG amplitude (i.e., EMG impulse) and centre of pressure amplitude during APAs to more thoroughly study the nature of adaptations in APAs and stability of the spine and trunk.

An important finding of this study was the difference in the recovery of EMG onset during APAs and RRs following the fourteen days of active recuperation. The EMG latencies during RRs have not shown any significant return to pre-BR levels. These differences might have been due to the specifics in the rehabilitation protocol. Unanticipated postural perturbations were applied primarily via lower limbs, but to a lesser extent using the arms. Future research should incorporate unanticipated postural perturbations applied via upper limbs or directly onto the torso. The APAs on the other hand showed a trend towards full recovery. This might additionally be due to the upper limb strengthening exercises.

The discrepancy between changes in EMG onset during APAs and RRs can also be attributed to their different physiological and neurological background. The APAs are centrally controlled, being primarily dependent on anticipation and prior experience. Their initiation is dependent on the anticipation of a forthcoming perturbation and its effect on posture and stability and not as a response to a perturbation in a feedback manner as is the case with RRs [12]. We can speculate that the timing of EMG onset during RRs is dependent on possible peripheral changes in sensory mechanisms such as changes in ligamentous apparatus of the spine due to changes in spinal curvature and length, atrophy of sensory enriched MU [17], and deprivation due to the lack of tonic stimulation [19] resulting in an altered sensory drive.

An alternative explanation of prolonged EMG latencies during RRs following BR can be based on the model proposed by Liebetrau et al. [40]. In this model, prolonged EMG latencies represent adjustments of the neuromuscular system to decreased trunk muscle activation amplitudes. These could be expected by generalizing findings from the observed decrease in lower limb power output following BR [41]. Based on these conclusions prolonged RRs might mirror changes in the neuromuscular strategies for preserving spinal stabilization. However, these adaptations suggest decreased capacity to preserve spinal stability under heavier load or fast occurring perturbations. To confirm this hypothesis, future research is needed. This should assess the muscle activation magnitude in order to assess the possible imbalances in force impulses of the trunk flexor and extensor muscles.

The above results represent an interesting insight into the effects of prolonged BR on muscle activation timing. However an important weakness of all BR studies, as well as of this one, is their small sample size due to the physical and social demands put on the participating subjects and high organizational demands. Future studies should continue measuring APAs and RRs in the context of BR enabling meta-analytical studies and consequently more valid information on the changes in APAs and RRs following prolonged physical inactivity.

## 5. Practical Relevance of the Results

BR has been shown to have a detrimental effect on specific aspects of the trunk stabilization functions (onset of EMG activity), especially during the unanticipated postural perturbations. As prolonged BR in the elderly is usually followed by a specialized rehabilitation, trunk stability must be a central concern to the therapists. These functions represent the base of any other rehabilitation activity and should be addressed accordingly. Future studies should assess the possible effect of vibration application [18, 42, 43], resistive and quick leg/arm movements [44], and unexpected perturbations to limb position on preserving trunk neuromuscular stabilization functions during and after BR. Special focus should be given to reactive trunk stabilizing actions, as these have been shown to be the most affected.

## Figures and Tables

**Table 1:** One-way repeated-measures ANOVA and corresponding post hoc *t*-tests for both tests of automatic (re)actions of the selected trunk muscles (obliquus externus (EO), obliquus internus (OI), lumbar erector spine (LE), and multifidus (MU)). Statistical significance of differences (*p*) and effect sizes (ES) are presented.

Muscle	RANOVA			Post hoc *t*-tests								
	PRE-POST0-POST14			PRE-POST0			PRE-POST14			POST0-POST14		
	*F*	*p*	ES	*t*	*p*	ES	*t*	*p*	ES	*t*	*p*	ES
Trunk anticipatory postural adjustments												
EO	1.874	.175	.135	1.139	.277	.312	1.773	.102	.456	0.782	.449	.220
IO	1.126	.341	.086	0.383	.708	.110	1.169	.265	.320	1.944	.076	.489
LE	3.761	.038	.239	0.931	.370	.259	2.445	.031	.577	2.223	.046	.540
MU	4.553	.021	.275	1.320	.211	.356	3.030	.010	.658	1.786	.099	.458

Trunk reflex reactions on mechanical perturbation												
EO	5.560	.015	.410	−1.994	.081	.576	−3.438	.009	.772	−1.172	.275	.383
IO	7.193	.005	.444	−3.610	.006	.769	−2.707	.024	.670	0.306	.766	.102
LE	10.439	.001	.487	−4.304	.001	.792	−2.963	.013	.666	1.496	.163	.411
MU	7.860	.003	.440	−3.501	.006	.742	−2.272	.046	.584	1.934	.082	.522
